# Lymphocyte Subsets and Cytokine Changes in Women With Gestational Diabetes Mellitus: A Systematic Review

**DOI:** 10.1155/jdr/3494697

**Published:** 2025-03-04

**Authors:** Wang Yu, Huang Miao, Yunhui Gong

**Affiliations:** ^1^Department of Gynecology and Obstetrics, West China Second University Hospital, Sichuan University, Chengdu, China; ^2^Key Laboratory of Birth Defects and Related Diseases of Women and Children, Ministry of Education, West China Second Hospital, Sichuan University, Chengdu, China

**Keywords:** cytokines, gestational diabetes marital, lymphocytes, systematic review

## Abstract

**Introduction:** Gestational diabetes mellitus (GDM) is a major health concern during pregnancy, affecting both the mother and the baby. Immune system alterations, particularly changes in lymphocyte subsets and cytokine profiles, have been associated with the pathophysiology of various metabolic disorders, including diabetes. This study is aimed at systematically reviewing the literature on the changes in lymphocyte subsets and cytokines in GDM.

**Methods:** In this systematic review, we applied specific criteria to select observational studies (such as case–controls, cross-sectionals, or cohorts) that focused on pregnant women. We performed an extensive search across electronic databases, including Web of Science, Scopus, PubMed, MEDLINE, Embase, Cochrane Central Register of Controlled Trials, and Google Scholar, from January 1, 2010, to March 20, 2024.

**Results:** A total of 19 articles, with 2517 participants (1128 with GDM and 1389 without GDM), were included in the qualitative synthesis. Due to high heterogeneity among the articles, a meta-analysis was not conducted. The studies assessed 35 different lymphocyte subsets or proportions. The most commonly assessed subsets were CD3+ T cell (five articles, mostly no difference between GDM and non-GDM), CD4+ T cell (five articles with contradictory results), CD8+ T cell (four articles with contradictory results), B cell and NK cell (three articles, mostly no difference between GDM and non-GDM), and Tregs (three articles with contradictory results). Additionally, 32 cytokines or proportions were assessed in the studies. The most commonly assessed cytokines were IL-6 (eight articles, higher or similar levels in GDM compared to non-GDM), TNF-*α* (seven articles, mostly higher or similar levels in GDM compared to non-GDM), IL-10 (six articles, mostly no difference between GDM and non-GDM), IL-2 (three articles, mostly no difference between GDM and non-GDM), and IFN-*γ* (three articles with contradictory results).

**Conclusion:** According to the results, there were no significant changes in CD3+ T cells, B cells, NK cells, IL-10, and IL-2 in GDM. However, the levels of IL-6 and TNF-*α* were higher or similar in GDM compared to non-GDM. The changes of other lymphocyte subsets and cytokines in GDM remained unclear.

## 1. Introduction

Gestational diabetes mellitus (GDM) is a type of diabetes that is first diagnosed in the second or third trimester of pregnancy. It is distinct from pre-existing Type 1 diabetes or Type 2 diabetes mellitus (T2DM) [1]. GDM is characterized by abnormal glucose tolerance, impaired insulin signaling, and insufficient insulin secretion [2]. The incidence of GDM is increasing rapidly, partially due to increasing body weight and maternal age [3].

The prevalence of GDM varies between 2% and 25% in different studies, depending on the screening criteria and the population characteristics [4–8]. GDM carries an increased risk of complications for both the mother and the fetus/child. Mothers with GDM face a higher risk of preeclampsia during pregnancy, as well as the development of T2DM and cardiovascular disease later in life. For the fetus/child, there is a higher chance of macrosomia, which is associated with T2DM, metabolic syndrome, cardiovascular disease, and even atopic/allergic diseases in later stages of life [4, 6, 8–10].

The underlying mechanisms of gestational diabetes are still not fully understood. In most women, the beta cells compensate by increasing insulin secretion to maintain normal blood sugar levels. However, in the case of GDM, this compensation is insufficient to counteract insulin resistance and hepatic glucose production [9]. Recent evidence indicates that GDM is not only characterized by insulin resistance and glucose intolerance but also by a chronic low-grade systemic inflammatory state and immune dysregulation, which disrupts the balance between Type 1 and Type 2 T helper cells [10, 11]. Chronic inflammation plays a crucial role in the pathophysiology and development of both diabetes mellitus and GDM [2]. Inflammatory cells such as neutrophils, monocytes, platelets, and lymphocytes, as well as various inflammatory markers or mediators, including cytokines like interleukin-1 (IL-1) and tumor necrosis factor-alpha (TNF-*α*), selectin E, C-reactive protein (CRP), and tissue plasminogen activator (t-PA), are elevated in individuals with diabetes [12, 13].

In women with GDM, these inflammatory factors are significantly higher compared to those with normal pregnancies. Some of these factors can serve as predictors of insulin sensitivity and GDM progression [14, 15]. A study conducted by Sheu et al. revealed that women with GDM exhibited a higher percentage of Th17 cells compared to a control group without GDM. Additionally, this study demonstrated that women with GDM had a greater proportion of proinflammatory cells during pregnancy compared to the control group [16]. Similarly, another study by Sargin et al. found that the number of leukocytes, neutrophils, and lymphocytes in the studied groups was significantly higher compared to that in the control group [17].

Inflammatory cells play a crucial role in systemic immune responses and various inflammatory diseases, such as cardiovascular diseases, acute kidney injury, and coronary artery transplantation [18]. In recent years, the absolute number and ratio of inflammatory cells have emerged as potential predictive indicators in inflammatory events. These indicators include the absolute lymphocyte count (ALC), absolute neutrophil count (ANC), neutrophil-to-lymphocyte ratio (NLR), monocyte-to-lymphocyte ratio (MLR), hemoglobin-to-platelet ratio (HPR), and platelet-to-lymphocyte ratio (PLR) [19, 20]. Their utilization as predictive indicators highlights their importance in assessing and monitoring inflammatory conditions.

Understanding the role of inflammation in GDM is crucial for improving risk assessment, diagnostic accuracy, and therapeutic approaches. By recognizing the role of inflammation in the pathophysiology of GDM, healthcare providers can better assess the risk and implement appropriate interventions and enhanced surveillance strategies. Integrating inflammatory markers into diagnostic algorithms can facilitate early diagnosis and intervention, ultimately lowering the risk of adverse outcomes for both the mother and child. Furthermore, addressing inflammation alongside traditional management strategies can enhance glycemic control and reduce rates of complications. Moreover, investigating the inflammatory basis of GDM offers valuable insights into its potential long-term health implications, allowing for early intervention and preventive measures targeting conditions like T2DM and cardiovascular disease. Understanding the inflammatory aspect of GDM not only enhances clinical care but also stimulates research and innovation, leading to more effective management and prevention strategies. Therefore, this study is aimed at systematically reviewing the literature on changes in lymphocyte subsets and cytokines in gestational diabetes.

## 2. Methods

### 2.1. Eligibility Criteria

In our systematic review, we included observational studies (such as case–controls, cross-sectionals, or cohorts) that examined changes in lymphocyte subsets and cytokines in pregnant women with GDM, using normoglycemic pregnancies as the control group. This comprehensive approach allowed us to explore the immune system changes associated with gestational diabetes in a rigorous manner.

#### 2.1.1. The Inclusion Criteria

(a) Participants: We included pregnant women diagnosed with GDM using any diagnostic criteria specified by the authors. (b) Study design: Our focus was on observational studies that involved healthy pregnant women without GDM or other clinical diseases, serving as a comparison or control group. (c) Laboratory methods: We considered studies that employed various assay methods. (d) Data: We included original reports with nonduplicated data that could be converted to mean ± SD. (e) Publication type: Our inclusion criteria encompassed original articles and articles in press. (f) Publication language: We restricted our search to full-text articles in the English language.

#### 2.1.2. The Exclusion Criteria

(a) Participants: Women with pregestational diabetes or other systemic illnesses affecting the levels of lymphocytes and cytokines, such as bronchial asthma and hypertension, and pregnant women with other types of diabetes mellitus (Type I and Type II) were excluded. (b) Study design: Studies conducted with other designs, such as reviews, experimental studies, qualitative studies, and case reports or series (that only reported lymphocyte and cytokine levels only in patients with GDM); animal studies; in vivo and in vitro studies; prediction studies that analyzed lymphocyte and cytokine levels before the onset of GDM as potential biomarkers for future development of the disease; proteomic studies (evaluating the function and structure of proteins); exclusively fetal and/or placental tissue studies (e.g., fetal biopsy or cord blood, placental biopsies); tissue-based studies; and mRNA expression studies were excluded. (c) Data: Studies that included pregnant women with all types of diabetes mellitus but did not provide separate data on GDM were excluded. (d) Publication type: Editorials, letters, conference papers, and comments were excluded. (e) Publication time: Articles published before 2010 due to potential changes in healthcare systems were excluded.

### 2.2. Source of Information

We performed an extensive search across multiple electronic databases, such as Web of Science, Scopus, PubMed, MEDLINE, Embase, and Google Scholar [21], until March 20, 2024. Furthermore, we broadened our search by examining the bibliographies of the studies incorporated in our analysis to ensure a comprehensive review of pertinent literature.

### 2.3. Search Strategy

Our search strategy was developed following the Peer-Reviewed Electronic Search Strategies Guideline [22] and tailored for each individual database search platform. This method was carefully designed to produce excellent outcomes. For specific details regarding the search strategies utilized, please refer to Appendix I.

### 2.4. Selection Process

**Figure 1:**
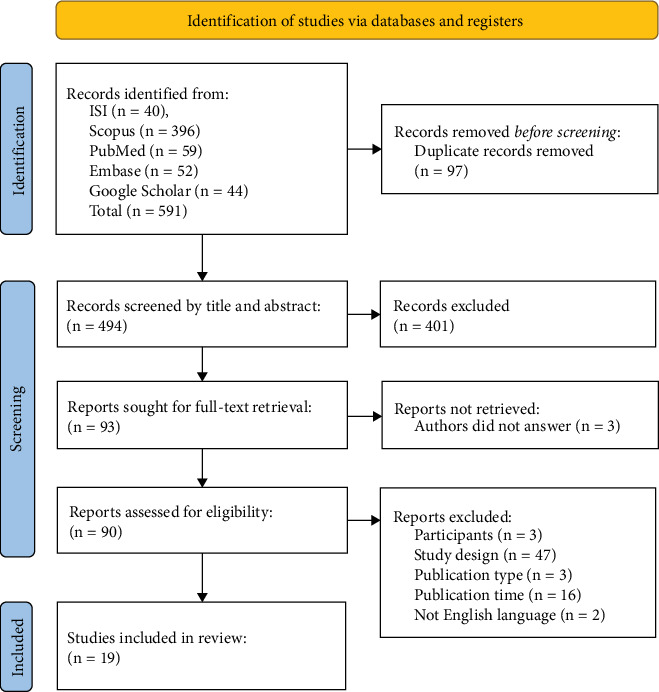
Flowchart of screening process.

Initially, one of the researchers imported all search findings from the databases into the EndNote Desktop software, eliminating any duplicates. Subsequently, two researchers independently assessed the titles and abstracts of articles based on predetermined eligibility criteria. In the event of any discrepancies in study selection, a thorough examination of the full text was conducted, with involvement from a third researcher if necessary. Attempts were made to acquire inaccessible articles and unpublished data by reaching out to the corresponding authors of eligible studies. The screening process was visually depicted using the Preferred Reporting Items for Systematic Reviews (PRISMA) 2020 flow diagram [23] (see Figure 1).

### 2.5. Data Collection Process

A data extraction form was developed, and consistency was ensured through a calibration exercise, where completed forms for the initial three articles were compared. Subsequently, two authors independently extracted data from all articles. Any discrepancies that emerged during this process were resolved through consensus discussions involving a third author. The form encompassed essential information such as study design, inclusion/exclusion criteria, sample size, diagnosis criteria for GDM, characteristics of study participants in the GDM and comparison/control groups (e.g., age, body mass index (BMI), blood sampling weeks of gestation, plasma glucose levels), sample type, assay method, mean percentage of lymphocyte subsets and subpopulations, mean cytokine levels, and statistical methods (e.g., risk ratio (95% CI) and adjusted statistics).

The primary outcome evaluated in this systematic review was the mean percentage of lymphocytes and cytokine levels.

### 2.6. Study Risk of Bias Assessment

We employed the Critical Appraisal Skills Programme (CASP) Case–Control Study Standard Checklist, the CASP Cohort Study Standard Checklist, and the Joanna Briggs Institute (JBI) Critical Appraisal Checklist for Analytical Cross-Sectional Studies to assess the quality and risk of bias in the included studies of present review.

The CASP Case–Control Study Standard Checklist consists of 11 criteria that cover various aspects of study design, conduct, and reporting. These criteria include a clear research question, appropriate selection of cases and controls, adequate sample size, valid measurement of exposure and outcome variables, consideration of confounding factors, appropriate statistical analysis, and clear presentation of results. Each criterion is scored as either “yes,” “no,” or “cannot tell” based on the information provided in the study [24].

The CASP Cohort Study Standard Checklist consists of 12 questions that help reviewers evaluate the internal validity of cohort studies. It examines key components such as the clarity of research question, appropriateness of the study design, selection of participants, measurement of exposure and outcomes, control for confounding variables, follow-up duration, and statistical analysis. Each criterion is scored as “yes,” “no,” or “cannot tell” based on the information provided in the study [25].

The JBI Critical Appraisal Checklist is specifically designed to assess the quality and risk of bias in analytical cross-sectional studies included in a review. It consists of eight questions that cover key methodological aspects such as study design, sampling methods, data collection procedures, statistical analysis, and potential sources of bias. Each criterion is scored as “yes,” “no,” “unclear,” or “not applicable” based on the information provided in the study [6].

Studies that did not meet more than half of the checklist items or were unclear were excluded from the analysis.

### 2.7. Data Synthesis

We performed a descriptive and qualitative synthesis of the study results for all included studies, which are presented in both narrative and tabular forms. Due to the high risk of publication bias and significant heterogeneity among the retrieved articles, we did not conduct meta-analysis.

## 3. Results

### 3.1. Literature Search

Initially, 591 articles were identified through the database search. Following the elimination of 97 duplicates, 494 titles and abstracts were screened, leading to the review of 93 full texts. Among these, 90 articles were assessed for eligibility criteria, leading to the inclusion of 19 articles in the study (refer to Figure 1).

### 3.2. Study Characteristics

Among the 19 selected studies, there were 3 cohort studies, 3 case–control studies, and 13 cross-sectional studies. Most of the studies employed nonrandom sampling methods. With the exception of four studies that had more than two groups, the remaining studies consisted of two groups: pregnant women with gestational diabetes (*n* = 1128) and healthy pregnant women (*n* = 1389). In most of the studies, diagnostic criteria for gestational diabetes included fasting blood glucose ≥ 5.1 mmol/L (92 mg/dL), 1-h blood glucose ≥ 10.0 mmol/L (180 mg/dL), or 2-h blood glucose ≥ 8.5 mmol/L (153 mg/dL) at 24 and 28 pregnancy weeks. The primary outcomes of interest in all studies were the frequency of lymphocyte subsets and cytokines during the first, second, or third trimester, and standard measurements were used to assess them (Table 1).

### 3.3. Lymphocyte Subsets in Women With GDM

Table 2 provides a summary of the assessment of 35 lymphocyte subsets or proportions in the studies. The most commonly assessed lymphocyte subsets were CD3+ T cell (five articles), CD4+ T cell (five articles), CD8+ T cell (four articles), B cell, NK cell, and regulatory T cells (Tregs) (three articles). Among the studies, one study reported lower level of CD3+ T cell in GDM than in non-GDM [32], while the other studies reported no differences in CD3+ T cell levels between the two groups [27, 33, 35, 37]. Pendeloski et al. [35] reported a higher level of CD4+ T cells in GDM than in non-GDM, while Hou and Li [32] and Friebe-Hoffmann et al. [35] reported lower levels of CD4+ T cells in GDM than in non-GDM. Zhu et al. [27] and Schober et al. [39] found similar levels of CD4+ T cells in GDM and non-GDM. The level of CD8+ T cells was reported as similar in GDM and non-GDM in two studies [27, 33], while two other studies reported higher levels in GDM than in non-GDM [32, 35]. For B cells, two studies reported similar levels in GDM and non-GDM [27, 33], while one study reported higher levels in GDM than in non-GDM [37]. Two studies found no difference in the level of NK cells between GDM and non-GDM [27, 33], while one study reported lower levels in GDM than in non-GDM [37]. Treg levels were reported as higher, lower, similar in GDM compared to non-GDM in the studies by Sifnaios et al. [1], Yang et al. [34], and Zhang et al. [26], respectively. The CD4+/CD8+ ratio was reported as lower in GDM compared to non-GDM in one study [32] and similar in another study [27]. Other lymphocyte subsets were only reported in one study.

### 3.4. Cytokine Changes in Women With GDM

Table 3 provides a summary of the assessment of 32 cytokines or proportions in the studies. The most commonly assessed cytokines were interleukin-6 (IL-6) (eight articles), TNF-*α* (seven articles), interleukin-10 (IL-10) (six articles), interleukin-2 (IL-2), and interferon-gamma (IFN-*γ*) (three articles). According to the results of four studies, the level of IL-6 was higher in GDM compared to non-GDM [27, 28, 32, 34], while the results of three studies showed no difference in the level of IL-6 between GDM and non-GDM [30, 41, 42]. In addition, in the study by Kuźmicki et al., patients from the GDM group had higher IL-6 at the first visit compared to the women with NGT, but there was no difference between the groups at the second visit [40]. The level of TNF-*α* was higher in GDM compared to non-GDM in four studies [32, 34, 36, 38], lower in one study [27], and similar between groups in two studies [30, 41]. The level of IL-10 was not different between GDM and non-GDM in four studies [27, 30, 36, 41], while it was lower in GDM than in non-GDM in two studies [32, 34]. The level of IL-2 was not different between GDM and non-GDM in two studies [30, 42], while it was lower in GDM than in non-GDM in one study [27]. Zhu et al. reported higher levels of IFN-*γ* in GDM than in non-GDM [27], while Hart et al. [29] reported lower levels, and Tagoma et al. [30] reported no difference in the level of IFN-*γ* between GDM and non-GDM. Other cytokines were only reported in one study.

## 4. Discussion

Researchers are increasingly focused on identifying biological markers to predict and diagnose GDM. GDM poses risks for mothers, fetuses, newborns, and adult offspring, making early detection crucial for effective care [43, 44]. Therefore, we aimed to investigate whether there were alterations in lymphocyte subsets and cytokines in women with GDM compared to those without GDM.

GDM is a common metabolic disorder that occurs during pregnancy [45–47]. Although its exact cause remains unclear, it is known to be associated with insulin resistance and *β*-cell dysfunction. Interestingly, during pregnancy, there is a natural development of insulin resistance starting around 12 weeks of gestation [37]. This insulin resistance gradually increases until full-term, resembling the pattern seen in T2DM. To compensate for the insulin resistance, *β*-cells produce higher levels of insulin to maintain stable blood glucose levels. However, in GDM pregnancies, the high insulin resistance, decreased insulin sensitivity, and impaired *β*-cell secretion lead to high levels of blood glucose [48, 49].

Immune dysfunction is assumed to play a critical role in insulin resistance and *β*-cell impairment. Several studies have demonstrated that T lymphocytes can infiltrate visceral adipose tissue (VAT) and produce proinflammatory cytokines, leading to the development of insulin resistance [39, 50, 51]. Regarding the immunological aspects, there is evidence suggesting that gestational diabetes leads to significant variations in immune parameters [44]. Elevated frequencies of CD3+ T cells and CD4+ T cell lymphocytes, as well as B cells, have been observed in individuals with GDM, indicating both cellular and humoral immune activation [39]. Two research teams [39, 52] examined TCD4+ and TCD8+ cells in peripheral blood samples from healthy individuals and those with GDM during the third trimester of pregnancy. Both studies revealed an elevated proportion of activated TCD4 cells and a decreased percentage of activated TCD8 cells in diabetic patients, with more pronounced alterations in those receiving insulin treatment. Additionally, Mahmoud et al. [39] noted an increased frequency of activated subsets of TCD4 cells and high levels of TCD4+ CD45RO and TCD4+ CD29+ cells, as well as high levels of TCD8+ CD45 lymphocytes, along with lower percentages of TCD4+ CD45RA cells, which may serve as suppressors. Conversely, Lobo et al. [52] observed compromised expression of costimulatory molecules in both TCD4 and TCD8 cells, implying a state of hyperactivation and a deficiency in suppressive mechanisms.

This systematic review revealed no statistically significant alterations observed in the levels of CD3+ T cells, B cells, and NK cells between individuals with GDM and those without the condition. This suggests a relative stability in these lymphocyte subsets despite the presence of GDM. However, there is uncertainty regarding other lymphocyte subsets, such as CD4+ T cells, CD8+ T cells, and Tregs. Inconsistencies in study results may be attributed to variations in methodologies, sample sizes, participant demographics, and diagnostic criteria for GDM across different studies. As a result, further research is needed to elucidate the potential alterations in these lymphocyte populations and their impact on immune function in the context of GDM. Clarifying these uncertainties is crucial for advancing our understanding of the immunological aspects of GDM.

Additionally, this study found no significant alterations in the levels of IL-10 and IL-2 between individuals with GDM and those without GDM. This suggests stability in the regulatory and T cell proliferation cytokines within the context of GDM. Considering that these cytokines contribute to the network of mediators implicated in insulin resistance [53], conducting new studies with well-defined criteria and larger participant cohorts may confirm the hypothesis that alterations in IL-10 and IL-2 cytokines are involved in the pathophysiology of GDM.

There were higher or similar levels of the inflammatory cytokines IL-6 and TNF-*α* in individuals with GDM compared to those without the condition. This suggests a potential proinflammatory state associated with GDM, which can negatively impact insulin secretion and sensitivity due to the influence of these inflammatory cytokines, particularly TNF-*α* [54]. Previous studies have also emphasized this issue, demonstrating increased levels of proinflammatory cytokines like TNF-*α* and IL-6, along with decreased levels of the anti-inflammatory cytokine IL-10, in women with GDM [55–58]. TNF-*α* and IL-6 are known to possess inflammatory properties and can induce insulin resistance, serving as significant links between obesity, diabetes, and chronic inflammation [53]. It was expected that patients with GDM would exhibit elevated levels of these cytokines compared to healthy pregnant women, although some studies have reported similar levels between GDM and non-GDM women [30, 41, 42]. This observation may be partially attributed to methodological differences, including variations in participant selection criteria, gestational age at sampling, and the lack of control for intervening factors. Studies have shown that mother's age and BMI are the most important intervening factors in changes in immune parameters. These factors can affect the level of cytokines and lymphocyte subtypes. Women with higher BMI tend to have higher levels of inflammatory cytokines such as IL-6 and TNF-*α*, which may influence the results of the present study [59].

The impact of IFN-*γ* in GDM remains uncertain due to inconsistencies across study findings. However, some researchers believe that certain cytokines, including IL-5, IFN-*γ*, and IL-18, could serve as key indicators for predicting the diagnosis of GDM, potentially functioning as biomarkers [27, 29]. These disparities may arise from methodological variations, sample characteristics, and the complex interplay of factors influencing cytokine regulation in GDM.

While this study provides valuable insights, it is important to acknowledge and address several limitations. There exists significant heterogeneity across studies, including variations in study designs, participant demographics, diagnostic criteria for GDM, and methodologies for assessing immune parameters. This heterogeneity can affect the generalizability and reliability of the findings. Potential biases, including publication bias and selection bias within individual studies, may also influence the overall conclusions. Additionally, variability in the definition and diagnosis of GDM across studies can introduce inconsistencies in participant classification. Furthermore, confounding factors such as pre-existing medical conditions, medication use, and lifestyle habits may influence immune parameters and their associations with GDM, posing challenges for controlling these variables across studies. Recognizing these limitations is crucial for accurately interpreting the results and identifying avenues for future research refinement.

Changes in immune parameters observed in this systematic review, such as increased levels of IL-6 and TNF-*α* or altered lymphocyte subsets, may play a significant role in the development of specific maternal and fetal complications associated with GDM. Elevated IL-6 and TNF-*α* levels have been implicated in insulin resistance and chronic low-grade inflammation, which could exacerbate hyperglycemia and predispose women with GDM to developing T2DM postpartum. Additionally, these inflammatory cytokines have been linked to endothelial dysfunction, potentially contributing to an increased risk of preeclampsia during pregnancy [60]. From a fetal perspective, the heightened inflammatory state in GDM may influence placental function and nutrient transfer, increasing the risk of macrosomia. For instance, studies suggest that the inflammatory milieu, marked by elevated IL-6 and TNF-*α* levels, may disrupt the insulin-like growth factor (IGF) signaling pathways critical for fetal growth regulation. Furthermore, dysregulation of Tregs, which were inconsistent across studies, could impair maternal–fetal tolerance, potentially increasing the risk of adverse immune-related outcomes, such as atopic disorders in the offspring [61, 62]. While these links are supported by existing literature, the heterogeneity of immune alterations observed in the reviewed studies underscores the need for further research. Future investigations should focus on longitudinal studies that correlate specific immune parameters with clinical outcomes, thereby clarifying their role in the pathophysiology of GDM-related complications.

### 4.1. Conclusion

According to the results, there were no significant changes in CD3+ T cell, B cell, NK cell, IL-10, and IL-2 in individuals with GDM. However, the levels of IL-6 and TNF-*α* were higher or similar in GDM compared to non-GDM individuals. The impact of other lymphocyte subsets and cytokines in GDM remains unclear.

### 4.2. Limitation

One of the main limitations of the present study is the insufficient attention to the time of sample collection (early or late pregnancy). The level of biomarkers may change during pregnancy and this issue can affect the results as an intervening factor. Future studies should consider the timed changes of the parameters.

## Figures and Tables

**Table 1:** Characteristics of included studies.

**Author(s), year**	**Country (city)**	**Study design**	**Study sample size**	**GDM diagnosis criteria**	**GDM group (** **m** **e** **a** **n** ± **SD)**					**Comparison/control group (** **m** **e** **a** **n** ± **SD)**					**Outcomes**	**Article's quality**
					**n**	**Age (year)**	**BMI**	**GW**	**Plasma glucose (mmol/L or mg/dL)**	**n**	**Age (year)**	**BMI**	**GW**	**Plasma glucose (mmol/L or mg/dL)**		
Zhang et al., 2024 [26]	China	Prospective study	44	Fasting blood glucose ≥ 5.1 mmol/L (92 mg/dL), 1-h blood glucose ≥ 10.0 mmol/L (180 mg/dL), or 2-h blood glucose ≥ 8.5 mmol/L (153 mg/dL) at 24 and 28 pregnancy weeks	21	29.06 ± 0.81	31.62 ± 0.42	37.38 ± 0.39	Fasting glucose (mmol/L) = 4.97 ± 0.05	23	29.41 ± 0.73	29.77 ± 0.55	37.14 ± 0.44	Fasting glucose (mmol/L) = 4.42 ± 0.1	There is no significant difference between the proportion of Tregs and mTregs in pregnant women with gestational diabetes mellitus (GDM) and healthy pregnant women during the third trimester	Moderate

Zhu et al., 2023 [27]	China	Population-based cohort	9725 (60 of them assessed for cells, B cells, and levels of several inflammatory cytokines)	75-g oral glucose tolerance test (OGTT): 0-h value ≥ 5.1 mmol/L, 1-h value ≥ 10.0 mmol/L, or/and 2-h value ≥ 8.5 mmol/L, between 24 and 28 weeks of gestation	30	30.0 (28.5–32.0)	22.9 (22.1–23.7)	26 (25–26)	FBG (mmol/L) = 4.9 (4.2–5.3) 1 h = 9.8 (8.3–10.9) 2 h = 8.5 (7.1–9.4)	30	29.0 (27.0–31.0)	22.2 (20.7–23.8)	26 (24–26)	FBG (mmol/L) = 4.2 (3.9–4.3) 1 h = 7.0 (5.6–8.0) 2 h = 6.3 (5.6–7.1)	There is no difference in the ratios of T cells (CD3+ T cells, CD4+ T cells, CD8+ T cells, and CD4+/CD8+ ratio), B cells, NK cells, and IL-10 between GDM cases and controls. However, higher levels of IL-4, IL-6, IFN-*γ*, and IL-17A were observed in the GDM cases compared to the controls, while TNF-*α* and IL-2 levels were lower in the GDM cases	Moderate

Srivastava et al., 2023 [28]	India	Cross-sectional	250 (50 nonpregnant women)	2-h plasma glucose ≥ 140 mg/mL	100	26.63 ± 9.6	25.27 ± 4.49	≤ 12, 13–26, and ≥ 27 weeks	2-h postprandial (mg/dL) = 149.49 ± 13.6	100	25.84 ± 4.02	23.97 ± 3.85	≤ 12, 13–26, and ≥ 27 weeks	2-h postprandial (mg/dL) = 106.76 ± 14.9	Serum IL-6 level was higher in GDM women compared to non-GDM pregnant women. Serum IL-6 levels were higher in the 1st trimester of GDM women compared to non-GDM pregnant women	Moderate

Hart et al., 2022 [29]	Canada	Cohort	124	Diagnostic ICD10-CA coding was used to identify cases of GDM (code O24.8)	31	18–35 = 18 (58.1%) ≥ 35 = 13 (41.9)	Normal = 11 (35.5) Overweight = 7 (22.6) Obese = 13 (41.9)	17–23	NR	93	18–35 = 54(58.1%) ≥ 35 = 39 (41.9%)	Normal = 33 (35.5) Overweight = 21 (22.6) Obese = 39 (41.9)	17–23	NR	Six cytokines' levels were identified in the GDM CART: interleukin-1 receptor antagonist (IL-1Ra) (cutoff: < 25 pg/mL), interleukin-5 (cutoff: ≥ 0.4 pg/mL), interferon-*γ* (cutoff: < 4.9 pg/mL), IL-1Ra (cutoff: ≥ 111 pg/mL), eotaxin (cutoff: ≥ 21 pg/mL), and interleukin-18 (cutoff: ≥ 155 pg/mL)	Moderate

Tagoma et al., 2022 [30]	Estonia	Cohort	213	A 2-h GTT test with 75-g glucose at gestational weeks 23–28	60	32.00 ± 5.35	Underweight = 3 (5%) Normal weight = 21 (35%) Overweight = 19 (32%) Obese = 17 (28%)	26.50 ± 1.63	Fasting glucose (mmol/L) = 5.1–0.44 1-h glucose (mmol/L) = 9.70–1.75 2-h glucose (mmol/L) = 7.25–1.35	153	30.00 ± 5.25	Underweight = 8 (5%) Normal weight = 68 (44%) Overweight = 50 (33%) Obese = 27 (18%)	26.29 ± 1.61	Fasting glucose (mmol/L) = 4.40–0.28 1-h glucose (mmol/L) = 6.70–1.46 2-h glucose (mmol/L) = 5.70–1.78	There were no differences between the two groups regarding cytokines (GM-CSF, IL-1*β*, sIL-1RI, IL-2, IL-2RA, IL-4, IL-5, IL-6, IL-7, IL-10, IL-12(p70), IL-13, IL-15, IL-17A, IL-17F, IL-21, IL-22, IL-23, IL-27, IFN-*γ*, sTNFRII, TNF*α*, TGF-*β*1, TGF-*β*2, and TGF-*β*3)	Moderate

Yousif et al., 2021 [31]	Iraq	Cross-sectional	150	75-g oral glucose tolerance test between the 24th and 28th weeks of pregnancy	75	31.42 ± 3.67	1st trimester = 28.78 ± 2.21 2nd and 3rd trimester = 30.08 ± 2.81	25.91 ± 1.39	97.30 ± 10.76	75	30.67 ± 3.75	1st trimester = 28.10 ± 2.09 2nd and 3rd trimester = 29.37 ± 2.75	26.13 ± 1.40	80.52 ± 6.43	In late second trimester and early third trimester of pregnancy, IL-1*β* concentrations increase in female with GDM compared with healthy pregnant female, while there were no differences between the two groups in the first trimester	Moderate

Hou and Li, 2020 [32]	China	Cross-sectional	111	75-g oral glucose tolerance test between the 24th and 28th weeks of pregnancy Fasting plasma glucose (FPG) 5.1 mmol/L, 1-h postload glucose 10.0 mmol/L, 2-h postload glucose 8.5 mmol/L	61 (normal mother age = 32 and abnormal mother age = 29)	Normal mother age = 28.27 ± 2.37 Abnormal mother age = 36.23 ± 3.03	Normal mother age = 24.12 ± 4.23 Abnormal mother age = 23.83 ± 3.48	NR	NR	50	30.23 ± 3.22	22.53 ± 2.99	NR	NR	Compared to the non-GDM group, the levels of TNF-*α* and IL-6 were significantly higher, while IL-10 was markedly lower in both AMA and NMA women with GDM, with the highest levels observed in the AMA group. Moreover, the levels of T leukomonocyte subgroups CD3 and CD4 and the CD4/CD8 ratio were greatly reduced in both AMA and NMA patients, especially in the AMA group, while the level of the T leukomonocyte subgroup CD8 was significantly higher in GDM patients	Moderate

Sifnaios et al., 2019 [1]	Greece	Cross-sectional	49	2-h 75-g oral glucose tolerance test during the 24th–26th gestation weeks Fasting plasma glucose (FPG) ≥ 92 mg/dL, 1-h postload glucose ≥ 180 mg/dL, 2-h postload glucose ≥ 153 mg/dL	26	34.0 ± 3.7	29.5 ± 11.8 kg/m^2^	28–34	NR	23	31.6 ± 5.2	26.2 ± 8.5 kg/m^2^	28–34	NR	T lymphocyte subsets during the third trimester of pregnancy showed the following: Th1: The proportion of CD3+CD4+IFN-*γ*+ cells was not different between the GDM and control groups Th2: In the GDM group, the proportion of IL-13-expressing cells (CD3+CD4+IL-13+) was higher compared to the control group Th17: The proportion of CD3+CD4+IL-17+ cells was higher in the GDM group compared to the control Treg: The proportion of CD3+CD4+IL-10+ cells was higher in the GDM group compared to the control	Moderate

Zhuang et al., 2019 [33]	China	Cross-sectional	292	75-g oral glucose tolerance test between the 24th and 28th weeks of pregnancy Fasting plasma glucose (FPG) 5.1 mmol/L, 1-h postload glucose 10.0 mmol/L, 2-h postload glucose 8.5 mmol/L	124	31.31 ± 5.12	NR	NR	NR	168	28.11 ± 3.75	NR	NR	NR	The percentage of B cells was higher in the GDM group than in the non-GDM group and the percentage of NK cells was lower. No differences were observed in the abundance of CD3+ T cell between the GDM and non-GDM groups. The percentage of B lymphocytes in the GDM group was higher than that in the non-GDM group	Moderate

Sheu et al., 2018 [16]	Australia	Prospective longitudinal case–control	120	Fasting plasma glucose ≥ 5.5 mmol/L and/or 2-h plasma glucose ≥ 8.0 mmol/L on a 2-h 75-g oral GTT	55	33.9 ± 3.6	25.1 ± 6.8	36–38	Fasting glucose (mmol/L) = 4.8 ± 0.6 1-h glucose (mmol/L) = 9.7 ± 1.5 2-h glucose (mmol/L) = 8.5 ± 1.4	65	33.2 ± 4.5	25.1 ± 5.5	36–38	Fasting glucose (mmol/L) = 4.3 ± 0.4 1-h glucose (mmol/L) = 6.7 ± 1.4 2-h glucose (mmol/L) = 5.4 ± 1.0	Women with GDM had a greater percentage of Th17 and Th17.1 cells compared with the control group. The median Th17:Treg, Th17.1:Treg, and Th1:Treg ratios were higher in women with GDM, with the proportion of Tregs being similar in both groups	Moderate

Yang et al., 2018 [34]	China	Cross-sectional	55	3-h oral glucose tolerance test Subjects with two or more values above 95, 180, 155, and 140 mg/dL for fasting, 1-, 2-, and 3-h postglucose load, respectively	21	32.1 ± 5.1	31.7 ± 3.1	10.8 ± 0.9	Fasting glucose (mg/dL) = 5.2 ± 0.4	34	32.5 ± 4.2	31.6 ± 3.7	10.3 ± 1.1	Fasting glucose (mg/dL) = 5.2 ± 0.4	In women with GDM compared to those without GDM, there were reduced levels of Tregs and elevated levels of serum interleukin-6 (IL-6) and tumor necrosis factor-alpha (TNF-alpha). Additionally, the Tregs in the GDM group showed reduced levels of transforming growth factor beta and IL-10 compared to the non-GDM group	High

Friebe-Hoffmann et al., 2017 [35]	Germany	Cross-sectional	119 (there were five groups)	Oral glucose tolerance test	18	33 (18–43)	NR	30 + 2	NR	24	34 (27–45)	NR	31	NR	*γδ* T cell levels were higher in women with GDM. On the other hand, the percentage of CD4+ T cells was reduced. There were no differences between the two groups regarding CD3+ T cells, CD8+ T cells, B cells, NK cells, NKT cells, DC, DC CD11c, and DC CD123	Moderate

Moreli et al., 2015 [36]	Brazil	Cross-sectional	192 (there were four groups)	Oral glucose tolerance test between the 24th and 28th gestational weeks	59	32.23 ± 6.34	36.63 ± 5.20	36	Glycemic mean = 107.68 ± 13.85 (mg/dL)	72	28.36 ± 6.66	33.28 ± 7.22	36	Glycemic mean = 81.08 ± 9.13 (mg/dL)	TNF-*α* plasma levels were higher in GDM patients compared to the normal group. IL-10 levels were not different between the two groups. The TNF-*α*/IL-10 ratio was higher in GDM patients compared to the normal group	Moderate

Pendeloski et al., 2015 [37]	Brazil	Case–control	50	2-h 75-g oral glucose tolerance test during the 24th–26th gestation weeks Fasting plasma glucose (FPG) ≥ 92 mg/dL, 1-h postload glucose ≥ 180 mg/dL, 2-h postload glucose ≥ 153 mg/dL	20	23–36	20–35	28–36	NR	30	19–46	20–33	28–36	NR	There were no significant differences in the frequency of CD3+, CD3+CD4+, CD3+CD8+ T lymphocytes, and CD4+HLA-DR+ T lymphocytes between the control and GDM groups. However, there was a higher frequency of CD4+ and CD8+ T cells expressing the early activation marker CD69+ in the GDM group GDM patients had higher proportions of CD3+CD4+-ICOS+, CD4+ICOS+HLA-DR+, CD4+CD28+HLA-DR+, and CD3+CD4+PD-1+ T cells, as well as a higher frequency of CD3+CD8+ICOS+, CD8+CD28+CD69+, CD8+CD28+HLA-DR+, and CD8+CTLA-4+HLA-DR+ T cells compared to healthy pregnant women On the other hand, GDM patients had a smaller number of CD3+CD4+CTLA-4+ T cells and a lower proportion of CD3+CD8+CTLA-4+ and CD8+ICOS+HLA-DR+ T cells than healthy controls	High

Noureldeen et al., 2014 [38]	Saudi Arabia	Cross-sectional	142	Fasting plasma glucose ≥ 7.0 mmol/L or plasma glucose after 2 h ≥ 7.8 mmol/L	71 (24 at the 2nd trimester and 47 at the 3rd trimester)	21–45	2nd trimester = 31.10 ± 1.066 3rd = 30.08 ± 0.736	2nd trimester = 21.25 ± 0.862 3rd = 33.85 ± 0.509	2nd trimester FBS (mg/dL) = 102.39 ± 5.381 3rd = 95.94 ± 3.150	71 (33 at the 2nd trimester and 38 at the 3rd trimester)	18–43	2nd trimester = 27.65 ± 1.284 3rd = 27.13 ± 0.817	2nd trimester = 20.59 ± 0.694 3rd = 32.16 ± 0.582	2nd trimester FBS (mg/dL) = 85.42 ± 1.581 3rd = 85.19 ± 1.719	In the second and third trimesters, the GDM group showed elevated mean values of TNF-*α* compared to the normal pregnant group	Moderate

Schober et al., 2014 [39]	Germany	Cross-sectional	125	2-h 75-g oral glucose tolerance test during the 24th–28th gestation weeks Fasting plasma glucose (FPG) ≥ 92 mg/dL, 1-h postload glucose ≥ 180 mg/dL, 2-h postload glucose ≥ 153 mg/dL	61 (21 dietary-adjusted GDM and 40 insulin dependent)	Dietary adjusted = 32 (25–43) Insulin dependent = 34 (22–43)	NR	Dietary adjusted = 39 (24–41) Insulin dependent = 36 (24–42)	NR	64	31 (21–44)	NR	37 (24–41)	NR	There were no differences in either the percentage of CD4+ T cells of total leukocytes or the percentage of CD4+CD127low+/−CD25+FoxP3+ Treg cells within the total CD4+ T cell pool between healthy pregnancies and pregnancies affected by dietary-adjusted or insulin-dependent GDM	Moderate

Kuźmicki et al., 2014 [40]	Poland	Cross-sectional	91	2-h 75-g oral glucose tolerance test during the 24th–28th gestation weeks Fasting plasma glucose (FPG) ≥ 100 mg/dL, 1-h postload glucose ≥ 180 mg/dL, 2-h postload glucose ≥ 140 mg/dL	GDM at the first visit (GDM1, *n* = 24) Normal glucose tolerance at the 1st visit and GDM at the 2nd visit (GDM2, *n* = 22)	GDM1 = 33 (31–34) GDM2 = 32 (28–34)	GDM1 = 27.3 (25.2–31.1) GDM2 = 28.0 (27.0–31.1)	GDM1 = 25 (24–27) GDM2 = 25 (24–28)	GDM1, fasting glucose (mmol/L) = 4.8 (4.0–5.2) GDM2, fasting glucose (mmol/L) = 4.6 (4.4–4.8)	45 (normal glucose tolerance at the first and second visits)	32 (28–35)	26.7 (22.9–29.4)	25 (24–27)	Fasting glucose (mmol/L) = 4.4 (4.1–4.6)	At the first visit, patients in the GDM1 group had higher IL-6 and sgp130 concentrations compared to women with NGT. There was no difference between the groups regarding IL-17, and the lowest levels of IL-23 were observed in the GDM1 group At the second visit, women with GDM2 had markedly higher sIL-6R levels compared to the NGT subjects. However, there was no difference between the groups regarding IL-6, IL-17, and IL-23	Moderate

Gueuvoghlanian-Silva et al., 2011 [41]	Brazil	Case–control	248	Fasting glucose ≥ 126 mg/dL (7.0 mm) and/or a 2-h post 75-g load ≥ 140 mg/dL (7.8 mm)	79	31.3 ± 6.0	58 (73.4)	32.2 ± 4.5	NR	169	29.1 ± 6.5	43 (28.3)	31.5 ± 4.0	NR	Cytokine levels (IL-10, IL-6, and TNF-A) were similar between the groups	High

Abdel Gader et al., 2011 [42]	Saudi Arabia	Cross-sectional	302 (52 GDM postdelivery)	Oral glucose tolerance test	150	28.8 + 6.3	34.4 + 5.9	38.1 ± 1.4	NR	100	28.0 + 7.5	30.6 + 4.6	38.2 ± 2.0	NR	No differences were noted in the mean values of IL-2, IL-6, and IL-8 between mothers with GDM and those with normal pregnancies	High

Abbreviations: BMI, body mass index; FPG, fasting plasma glucose; GDM, gestational diabetes mellitus; GW, gestation week; IFN, interferon; IL, interleukin; *n*, number of participants in each group; NR, not reported; SD, standard deviation; TNF, tumor necrosis factor.

**Table 2:** The literature results regarding the lymphocyte subset changes in gestational diabetes mellitus.

**Lymphocyte subsets**	**Higher (↑) in GDM than non-GDM**	**Lower (↓) in GDM than non-GDM**	**No difference (↔) between GDM and non-GDM**
CD3+ T cell		Hou and Li, 2020 [32]	Zhu et al., 2023 [27], Zhuang et al., 2019 [33], Friebe-Hoffmann et al., 2017 [35], Pendeloski et al., 2015 [37]
CD4+ T cell	Pendeloski et al., 2015 [37]	Hou and Li, 2020 [32], Friebe-Hoffmann et al., 2017 [35]	Zhu et al., 2023 [27], Schober et al., 2014 [39]
CD8+ T cell	Hou and Li, 2020 [32], Pendeloski et al., 2015 [37]		Zhu et al., 2023 [27], Friebe-Hoffmann et al., 2017 [35]
CD3+CD4+			Pendeloski et al., 2015 [37]
CD3+CD8+			Pendeloski et al., 2015 [37]
CD4+/CD8+ ratio		Hou and Li, 2020 [32]	Zhu et al., 2023 [27]
CD4+CD127low+/−CD25+FoxP3+ Treg			Schober et al., 2014 [39]
CD4+HLA-DR+ T	Pendeloski et al., 2015 [37]		Pendeloski et al., 2015 [37]
CD3+CD4+-ICOS+	Pendeloski et al., 2015 [37]		
CD4+ICOS+HLA-DR+	Pendeloski et al., 2015 [37]		
CD4+CD28+HLA-DR+	Pendeloski et al., 2015 [37]		
CD3+CD4+PD-1+	Pendeloski et al., 2015 [37]		
CD3+CD8+ICOS+	Pendeloski et al., 2015 [37]		
CD8+CD28+CD69+	Pendeloski et al., 2015 [37]		
CD8+CD28+HLA-DR+	Pendeloski et al., 2015 [37]		
CD8+CTLA-4+HLA-DR+	Pendeloski et al., 2015 [37]		
CD3+CD4+CTLA-4+		Pendeloski et al., 2015 [37]	
CD3+CD8+CTLA-4+		Pendeloski et al., 2015 [37]	
CD8+-ICOS+HLA-DR+ T		Pendeloski et al., 2015 [37]	
B cell	Zhuang et al., 2019 [33]		Zhu et al., 2023 [27], Friebe-Hoffmann et al., 2017 [35]
NK cell		Zhuang et al., 2019 [33]	Zhu et al., 2023 [27], Friebe-Hoffmann et al., 2017 [35]
NKT cell			Friebe-Hoffmann et al., 2017 [35]
DC			Friebe-Hoffmann et al., 2017 [35]
DC CD11c			Friebe-Hoffmann et al., 2017 [35]
DCCD123			Friebe-Hoffmann et al., 2017 [35]
Tregs	Sifnaios et al., 2019 [1]	Yang et al., 2018 [34]	Zhang et al., 2024 [26]
mTregs			Zhang et al., 2024 [26]
Th1			Sifnaios et al., 2019 [1]
Th2	Sifnaios et al., 2019 [1]		
Th17	Sifnaios et al., 2019 [1], Sheu et al., 2018 [16]		
Th17.1	Sheu et al., 2018 [16]		
Th1:Treg	Sheu et al., 2018 [16]		
Th17:Treg	Sheu et al., 2018 [16]		
Th17.1:Treg	Sheu et al., 2018 [16]		
*γδ* T cell	Friebe-Hoffmann et al., 2017 [35]		

Abbreviation: GDM, gestational diabetes mellitus.

**Table 3:** The literature results regarding the cytokine changes in gestational diabetes mellitus.

**Cytokines**	**Higher (↑) in GDM than non-GDM**	**Lower (↓) in GDM than non-GDM**	**No difference (↔) between GDM and non-GDM**
IL-1Ra	Hart et al., 2022 [29]^a^		
IL-1*β*	Yousif et al., 2021 [31]		Tagoma et al., 2022 [30]
sIL-1RI			Tagoma et al., 2022 [30]
IL-2		Zhu et al., 2023 [27]	Tagoma et al., 2022 [30], Abdel Gader et al., 2011 [42]
IL-2RA			Tagoma et al., 2022 [30]
IL-4	Zhu et al., 2023 [27]		Tagoma et al., 2022 [30]
IL-5	Hart et al., 2022 [29]		Tagoma et al., 2022 [30]
IL-6	Zhu et al., 2023 [27], Srivastava et al. 2023 [28], Hou and Li, 2020 [32], Yang et al., 2018 [34], Kuźmicki et al., 2014 [40]		Tagoma et al., 2022 [30], Kuźmicki et al., 2014 [40], Gueuvoghlanian-Silva et al., 2011 [41], Abdel Gader et al., 2011 [42]
sIL-6R	Kuźmicki et al., 2014 [40]		
IL-7			Tagoma et al., 2022 [30]
IL-8			Abdel Gader et al., 2011 [42]
IL-10		Hou and Li, 2020 [32], Yang et al., 2018 [34]	Zhu et al., 2023 [27], Tagoma et al., 2022 [30], Moreli et al., 2015 [36], Gueuvoghlanian-Silva et al., 2011 [41]
IL-12(p70)			Tagoma et al., 2022 [30]
IL-13			Tagoma et al., 2022 [30]
IL-15			Tagoma et al., 2022 [30]
IL-17			Kuźmicki et al., 2014 [40]
IL-17A	Zhu et al., 2023 [27]		Tagoma et al., 2022 [30]
IL-17F			Tagoma et al., 2022 [30]
IL-18	Hart et al., 2022 [29]		
IL-21			Tagoma et al., 2022 [30]
IL-22			Tagoma et al., 2022 [30]
IL-23		Kuźmicki et al., 2014 [40]	Tagoma et al., 2022 [30], Kuźmicki et al., 2014 [40]
IL-27			Tagoma et al., 2022 [30]
TNF-*α*	Hou and Li, 2020 [32], Yang et al., 2018 [34], Moreli et al., 2015 [36], Noureldeen et al., 2014 [38]	Zhu et al., 2023 [27]	Tagoma et al., 2022 [30], Gueuvoghlanian-Silva et al., 2011 [41]
IFN-*γ*	Zhu et al., 2023 [27]	Hart et al., 2022 [29]	Tagoma et al., 2022 [30]
sTNFRII			Tagoma et al., 2022 [30]
TGF		Yang et al., 2018 [34]	
TGF-*β*1			Tagoma et al., 2022 [30]
TGF-*β*2			Tagoma et al., 2022 [30]
TGF-*β*3			Tagoma et al., 2022 [30]
TNF-a/IL-10 ratio	Moreli et al., 2015 [36]		
Eotaxin	Hart et al., 2022 [29]		

Abbreviation: GDM, gestational diabetes mellitus.

^a^In Hart et al.'s (2022) [29] study, of the 42 cytokines that were identified to be present within the maternal plasma at 17–23 gestational weeks, 39 were measured, only the significant ones mentioned in the table.

## Data Availability

The data that support the findings of this study are available from the corresponding author upon reasonable request.

## Nomenclature

Tregs: T regulatory cells
IL-6: interleukin-6
IL-10: interleukin-10
IL-2: interleukin-2
GDM: gestational diabetes mellitus
PRISMA: Preferred Reporting Items for Systematic Reviews and Meta-Analyses
CASP: Critical Appraisal Skills Programme
JBI: Joanna Briggs Institute
CD3+ T: cluster of differentiation 3 positive T cells
CD4+ T: cluster of differentiation 4 positive T helper cells
CD8+ T: cluster of differentiation 8 positive cytotoxic T cells
B cell: B lymphocyte cells
NK cell: natural killer cells
TNF-*α*: tumor necrosis factor-alpha
IFN-*γ*: interferon-gamma

## Appendix I

We developed search strategies tailored to each database search engine to optimize the relevance and precision of our results.

All databases were searched on March 20, 2024.

Web of Science (filters∗: English; article; early access):

*(ALL = (Lymphocyte) OR ALL = (“T Cell”) OR ALL = (“Thymus Cell”) OR ALL = (“T Regulatory”) OR ALL = (“Regulatory T”) OR ALL = (Treg) OR ALL = (“T Helper”) OR ALL = (“Helper T”) OR ALL = (“Th Cell”) OR ALL = (“T Cytotoxic”) OR ALL = (“Cytotoxic T”) OR ALL = (“TC Cell”) OR ALL = (“Naive T”) OR ALL = (“TN Cell”) OR ALL = (“Killer T”) OR ALL = (“NKT Cell”) OR ALL = (“T Memory”) OR ALL = (“Memory T”) OR ALL = (“B Cell”) OR ALL = (“Bursa-Dependent”) OR ALL = (“Bursa-derived”) OR ALL = (“Regulatory B”) OR ALL = (“B Regulatory”) OR ALL = (“Breg”) OR ALL = (“Memory B”) OR ALL = (“B Memory”) OR ALL = (“Innate Lymphoid Cell”) OR ALL = (“ILCs”) OR ALL = (“Killer Cell”) OR ALL = (“NK Cell”) OR ALL = (CD3+) OR ALL = (CD4+) OR ALL = (CD8+) OR ALL = (CD16+) OR ALL = (CD19+) OR ALL = (CD25+) OR ALL = (CD29+) OR ALL = (CD45+) OR ALL = (CD54+) OR ALL = (CD56+) OR ALL = (HLA-DR)) AND (ALL = (Cytokine) OR ALL = (“Forkhead Box P3”) OR ALL = (FOXP3+) OR ALL = (“Transforming Growth Factor”) OR ALL = (TGF) OR ALL = (“Tumor Necrosis Factor”) OR ALL = (TNF) OR ALL = (Lymphotoxin) OR ALL = (LT) OR ALL = (Interferon) OR ALL = (IFN) OR ALL = (“Interleukin-1”) OR ALL = (“IL-1”) OR ALL = (“Interleukin-2”) OR ALL = (“IL-2”) OR ALL = (“Interleukin-4”) OR ALL = (“IL-4”) OR ALL = (“Interleukin-5”) OR ALL = (“IL-5”) OR ALL = (“Interleukin-6”) OR ALL = (“IL-6”) OR ALL = (“Interleukin-10”) OR ALL = (“IL-10”) OR ALL = (“Interleukin-13”) OR ALL = (“IL-13”) OR ALL = (“Interleukin-17”) OR ALL = (“IL- 17”) OR ALL = (“Interleukin-18”) OR ALL = (“IL- 18”) OR ALL = (“Interleukin-35”) OR ALL = (“IL-35”)) AND (TS = (“Gestational Diabetes”) OR TS = (GDM) OR TS = (“Pregnancy-induced Diabetes”) OR TS = (“Glucose Intolerance in Pregnancy”) OR TS = (“Hyperglycemia in Pregnancy”)) NOT (TS = (Intervention*⁣^∗^*) OR TS = (Trial) OR TS = (Experiment*⁣^∗^*) OR TS = (Qualitative) OR TS = (Review) OR TS = (“Case report”) OR TS = (Editorial) OR TS = (Letter) OR TS = (Conference) OR TS = (Comment) OR TS = (Animal) OR TS = (Mouse) OR TS = (Mice))*

Scopus (filters⁣^∗^: English; article):

*((ALL(Lymphocyte) OR ALL(“T Cell”) OR ALL(“Thymus Cell”) OR ALL(T W/0 Regulatory) OR ALL(Treg) OR ALL(T W/0 Helper) OR ALL(“Th Cell”) OR ALL(T W/0 Cytotoxic) OR ALL(“TC Cell”) OR ALL(“Na?ve T”) OR ALL(“TN Cell”) OR ALL(“Killer T”) OR ALL(“NKT Cell”) OR ALL(T W/0 Memory) OR ALL(“B Cell”) OR ALL(“Bursa-Dependent”) OR ALL(“Bursa-derived”) OR ALL(Regulatory W/0 B) OR ALL(“Breg”) OR ALL(Memory W/0 B) OR ALL(“Innate Lymphoid Cell”) OR ALL(“ILCs”) OR ALL(“Killer Cell”) OR ALL(“NK Cell”) OR ALL(CD3+) OR ALL(CD4+) OR ALL(CD8+) OR ALL(CD16+) OR ALL(CD19+) OR ALL(CD25+) OR ALL(CD29+) OR ALL(CD45+) OR ALL(CD54+) OR ALL(CD56+) OR ALL(HLA-DR)) AND (ALL(Cytokine) OR ALL(“Forkhead Box P3”) OR ALL(FOXP3+) OR ALL(“Transforming Growth Factor”) OR ALL(TGF) OR ALL(“Tumor Necrosis Factor”) OR ALL(TNF) OR ALL(Lymphotoxin) OR ALL(LT) OR ALL(Interferon) OR ALL(IFN) OR ALL(“Interleukin-1”) OR ALL(“IL-1”) OR ALL(“Interleukin-2”) OR ALL(“IL-2”) OR ALL(“Interleukin-4”) OR ALL(“IL-4”) OR ALL(“Interleukin-5”) OR ALL(“IL-5”) OR ALL(“Interleukin-6”) OR ALL(“IL-6”) OR ALL(“Interleukin-10”) OR ALL(“IL-10”) OR ALL(“Interleukin-13”) OR ALL(“IL-13”) OR ALL(“Interleukin-17”) OR ALL(“IL- 17”) OR ALL(“Interleukin-18”) OR ALL(“IL- 18”) OR ALL(“Interleukin-35”) OR ALL(“IL-35”)) AND (TITLE-ABS-KEY(“Gestational Diabetes”) OR TITLE-ABS-KEY(GDM) OR TITLE-ABS-KEY(“Pregnancy-induced Diabetes”) OR TITLE-ABS-KEY(“Glucose Intolerance in Pregnancy”) OR TITLE-ABS-KEY(“Hyperglycemia in Pregnancy”))) AND NOT TITLE-ABS-KEY(Intervention*⁣^∗^*) OR TITLE-ABS-KEY(Trial) OR TITLE-ABS-KEY(Experiment*⁣^∗^*) OR TITLE-ABS-KEY(Qualitative) OR TITLE-ABS-KEY(Review) OR TITLE-ABS-KEY(“Case report”) OR TITLE-ABS-KEY(Editorial) OR TITLE-ABS-KEY(Letter) OR TITLE-ABS-KEY(Conference) OR TITLE-ABS-KEY(Comment) OR TITLE-ABS-KEY(Animal) OR TITLE-ABS-KEY(Mouse) OR TITLE-ABS-KEY(Mice)*

PubMed (filters⁣^∗^: English):

*(Lymphocyte OR ‘T Cell' OR ‘Thymus Cell' OR ‘T Regulatory' OR ‘Regulatory T' OR Treg OR ‘T Helper' OR ‘Helper T' OR ‘Th Cell' OR ‘T Cytotoxic' OR ‘Cytotoxic T' OR ‘TC Cell' OR ‘Naive T' OR ‘TN Cell' OR ‘Killer T' OR ‘NKT Cell' OR ‘T Memory' OR ‘Memory T' OR ‘B Cell' OR ‘Bursa-Dependent' OR ‘Bursa-derived' OR ‘Regulatory B' OR ‘B Regulatory' OR Breg OR ‘Memory B' OR ‘B Memory' OR ‘Innate Lymphoid Cell' OR ‘ILCs' OR ‘Killer Cell' OR ‘NK Cell' OR CD3+ OR CD4+ OR CD8+ OR CD16+ OR CD19+ OR CD25+ OR CD29+ OR CD45+ OR CD54+ OR CD56+ OR HLA-DR) AND (Cytokine OR ‘Forkhead Box P3' OR FOXP3+ OR ‘Transforming Growth Factor' OR TGF OR ‘Tumor Necrosis Factor' OR TNF OR Lymphotoxin OR LT OR Interferon OR IFN OR ‘Interleukin-1' OR ‘IL-1' OR ‘Interleukin-2' OR ‘IL-2' OR ‘Interleukin-4' OR ‘IL-4' OR ‘Interleukin-5' OR ‘IL-5' OR ‘Interleukin-6' OR ‘IL-6' OR ‘Interleukin-10' OR ‘IL-10' OR ‘Interleukin-13' OR ‘IL-13' OR ‘Interleukin-17' OR ‘IL- 17' OR ‘Interleukin-18' OR ‘IL- 18' OR ‘Interleukin-35' OR ‘IL-35') AND (‘Gestational Diabetes'[Title/Abstract] OR GDM[Title/Abstract] OR ‘Pregnancy-induced Diabetes'[Title/Abstract] OR ‘Glucose Intolerance in Pregnancy'[Title/Abstract] OR ‘Hyperglycemia in Pregnancy'[Title/Abstract]) NOT (Intervention*⁣^∗^* [Title/Abstract] OR Trial[Title/Abstract] OR Experiment*⁣^∗^* [Title/Abstract] OR Qualitative[Title/Abstract] OR Review[Title/Abstract] OR ‘Case report'[Title/Abstract] OR Editorial[Title/Abstract] OR Letter[Title/Abstract] OR Conference[Title/Abstract] OR Comment[Title/Abstract] OR Animal[Title/Abstract] OR Mouse[Title/Abstract] OR Mice[Title/Abstract])'*

Embase (filters⁣^∗^: English; article; article in press; preprint; female):

*(‘lymphocyte'/exp OR lymphocyte OR ‘t cell'/exp OR ‘t cell' OR ‘thymus cell'/exp OR ‘thymus cell' OR ‘t regulatory' OR ‘regulatory t' OR ‘treg'/exp OR treg OR ‘t helper' OR ‘helper t' OR ‘th cell' OR ‘t cytotoxic' OR ‘cytotoxic t' OR ‘tc cell' OR ‘na?ve t' OR ‘tn cell' OR ‘killer t' OR ‘nkt cell'/exp OR ‘nkt cell' OR ‘t memory' OR ‘memory t' OR ‘b cell'/exp OR ‘b cell' OR ‘bursa-dependent' OR ‘bursa-derived' OR ‘regulatory b' OR ‘b regulatory' OR ‘breg'/exp OR breg OR ‘memory b' OR ‘b memory' OR ‘innate lymphoid cell'/exp OR ‘innate lymphoid cell' OR ‘ilcs' OR ‘killer cell'/exp OR ‘killer cell' OR ‘nk cell'/exp OR ‘nk cell' OR ‘cd3+'/exp OR cd3+ OR ‘cd4+'/exp OR cd4+ OR ‘cd8+'/exp OR cd8+ OR cd16+ OR cd19+ OR cd25+ OR cd29+ OR cd45+ OR cd54+ OR cd56+ OR ‘hla dr') AND (‘cytokine'/exp OR cytokine OR ‘forkhead box p3' OR foxp3+ OR ‘transforming growth factor'/exp OR ‘transforming growth factor' OR ‘tgf'/exp OR tgf OR ‘tumor necrosis factor'/exp OR ‘tumor necrosis factor' OR ‘tnf'/exp OR tnf OR ‘lymphotoxin'/exp OR lymphotoxin OR lt OR ‘interferon'/exp OR interferon OR ‘ifn'/exp OR ifn OR ‘interleukin-1'/exp OR ‘interleukin-1' OR ‘il-1'/exp OR ‘il-1' OR ‘interleukin-2'/exp OR ‘interleukin-2' OR ‘il-2'/exp OR ‘il-2' OR ‘interleukin-4'/exp OR ‘interleukin-4' OR ‘il-4'/exp OR ‘il-4' OR ‘interleukin-5'/exp OR ‘interleukin-5' OR ‘il-5'/exp OR ‘il-5' OR ‘interleukin-6'/exp OR ‘interleukin-6' OR ‘il-6'/exp OR ‘il-6' OR ‘interleukin-10'/exp OR ‘interleukin-10' OR ‘il-10'/exp OR ‘il-10' OR ‘interleukin-13'/exp OR ‘interleukin-13' OR ‘il-13'/exp OR ‘il-13' OR ‘interleukin-17'/exp OR ‘interleukin-17' OR ‘il- 17'/exp OR ‘il- 17' OR ‘interleukin-18'/exp OR ‘interleukin-18' OR ‘il- 18'/exp OR ‘il- 18' OR ‘interleukin-35'/exp OR ‘interleukin-35' OR ‘il-35'/exp OR ‘il-35') AND (‘gestational diabetes':ti,ab,kw OR gdm:ti,ab,kw OR ‘pregnancy-induced diabetes':ti,ab,kw OR ‘glucose intolerance in pregnancy':ti,ab,kw OR ‘hyperglycemia in pregnancy':ti,ab,kw) NOT (intervention*⁣^∗^* :ti,ab,kw OR trial:ti,ab,kw OR experiment*⁣^∗^* :ti,ab,kw OR qualitative:ti,ab,kw OR review:ti,ab,kw OR ‘case report':ti,ab,kw OR editorial:ti,ab,kw OR letter:ti,ab,kw OR conference:ti,ab,kw OR comment:ti,ab,kw OR animal:ti,ab,kw OR mouse:ti,ab,kw OR mice:ti,ab,kw)*

Google Scholar (filters: none):

*allintitle: Lymphocyte “Gestational Diabetes”*

*allintitle: Cytokine “Gestational Diabetes”*

⁣^∗^After retrieving search results, filters were applied using the filter panel on the results page.

## References

[1] Sifnaios E., Mastorakos G., Psarra K., Panagopoulos N. (2019). Gestational diabetes and T-cell (Th1/Th2/Th17/Treg) immune profile. *In Vivo*.

[2] Ahmadi M., Maleknia M., Javan M., Abdolaliyan M., Golshahi F., Vahabi E. (2022). Investigating hematological inflammatory indices in women with gestational diabetes. *The Iranian Journal of Obstetrics, Gynecology and Infertility*.

[3] Hedderson M., Gunderson E., Ferrara A. (2010). Gestational weight gain and risk of gestational diabetes mellitus. *Obstetrics and Gynecology*.

[4] Aydın H., Çelik Ö., Yazıcı D. (2019). Prevalence and predictors of gestational diabetes mellitus: a nationwide multicentre prospective study. *Diabetic Medicine*.

[5] Eades C., Cameron D., Evans J. (2017). Prevalence of gestational diabetes mellitus in Europe: a meta-analysis. *Diabetes Research and Clinical Practice*.

[6] Gao C., Sun X., Lu L., Liu F., Yuan J. (2019). Prevalence of gestational diabetes mellitus in mainland China: a systematic review and meta-analysis. *Journal of Diabetes Investigation*.

[7] Karasneh R., Migdady F., Alzoubi K., Al-Azzam S., Khader Y., Nusair M. (2021). Trends in maternal characteristics, and maternal and neonatal outcomes of women with gestational diabetes: a study from Jordan. *Annals of Medicine and Surgery*.

[8] Zhu Y., Zhang C. (2016). Prevalence of gestational diabetes and risk of progression to type 2 diabetes: a global perspective. *Current Diabetes Reports*.

[9] Correa P., Vargas J., Sen S., Illanes S. (2014). Prediction of gestational diabetes early in pregnancy: targeting the long-term complications. *Gynecologic and Obstetric Investigation*.

[10] Lekva T., Norwitz E., Aukrust P., Ueland T. (2016). Impact of systemic inflammation on the progression of gestational diabetes mellitus. *Current Diabetes Reports*.

[11] Richardson A., Carpenter M. (2007). Inflammatory mediators in gestational diabetes mellitus. *Obstetrics and Gynecology Clinics of North America*.

[12] Cinkajzlová A., Anderlová K., Šimják P. (2020). Subclinical inflammation and adipose tissue lymphocytes in pregnant females with gestational diabetes mellitus. *The Journal of Clinical Endocrinology & Metabolism*.

[13] Jensen M., Barrett H., Peek M., Gibson P., Murphy V. (2021). Maternal asthma and gestational diabetes mellitus: exploration of potential associations. *Obstetric Medicine*.

[14] Al-Ofi E., Alrafiah A., Maidi S., Almaghrabi S., Hakami N. (2021). Altered expression of angiogenic biomarkers in pregnancy associated with gestational diabetes. *International Journal of General Medicine*.

[15] Bogdanet D., Reddin C., Murphy D. (2021). Emerging protein biomarkers for the diagnosis or prediction of gestational diabetes—a scoping review. *Journal of Clinical Medicine*.

[16] Sheu A., Chan Y., Ferguson A. (2018). A proinflammatory CD4+ T cell phenotype in gestational diabetes mellitus. *Diabetologia*.

[17] Sargın M., Yassa M., Taymur B., Celik A., Ergun E., Tug N. (2016). Neutrophil-to-lymphocyte and platelet-to-lymphocyte ratios: are they useful for predicting gestational diabetes mellitus during pregnancy?. *Therapeutics and Clinical Risk Management*.

[18] Larmann J., Handke J., Scholz A. S. (2020). Preoperative neutrophil to lymphocyte ratio and platelet to lymphocyte ratio are associated with major adverse cardiovascular and cerebrovascular events in coronary heart disease patients undergoing non-cardiac surgery. *BMC Cardiovascular Disorders*.

[19] Furuncuoğlu Y., Tulgar S., Dogan A., Cakar S., Tulgar Y., Cakiroglu B. (2016). How obesity affects the neutrophil/lymphocyte and platelet/lymphocyte ratio, systemic immune-inflammatory index and platelet indices: a retrospective study. *European Review for Medical and Pharmacological Sciences*.

[20] Zare H., Nouri N., Peracheh M., Kikha N., Ghasemi M. (2021). Neutrophil to lymphocytes ratio and platelets to lymphocytes ratio in pre-eclampsia and normal pregnancy: a case-control study. *Iranian Journal of Obstetrics, Gynecology and Infertility*.

[21] Bramer W. M., Rethlefsen M. L., Kleijnen J., Franco O. H. (2017). Optimal database combinations for literature searches in systematic reviews: a prospective exploratory study. *Systematic Reviews*.

[22] Sampson M., McGowan J., Cogo E., Grimshaw J., Moher D., Lefebvre C. (2009). An evidence-based practice guideline for the peer review of electronic search strategies. *Journal of Clinical Epidemiology*.

[23] Page M. J., McKenzie J. E., Bossuyt P. M. (2021). The PRISMA 2020 statement: an updated guideline for reporting systematic reviews. *International Journal of Surgery*.

[24] https://casp-uk.net/casp-tools-checklists/.

[25] https://casp-uk.net/casp-tools-checklists/.

[26] Zhang Y. N., Wu Q., Deng Y. H. (2024). Phenotypic characterisation of regulatory T cells in patients with gestational diabetes mellitus. *Scientific Reports*.

[27] Zhu H., Zhao Z., Xu J. (2023). Comprehensive landscape of the T and B-cell repertoires of newly diagnosed gestational diabetes mellitus. *Genomics*.

[28] Srivastava N., Singh K., Singh N., Mahdi A. A. (2023). Association between serum interleukin-6, leptin and insulin in gestational diabetes mellitus – a cross- sectional study. *Journal of Diabetes and Metabolic Disorders*.

[29] Hart P. M., Stephenson N. L., Scime N. V., Tough S. C., Slater D. M., Chaput K. H. (2022). Second trimester cytokine profiles associated with gestational diabetes and hypertensive disorders of pregnancy. *PLoS One*.

[30] Tagoma A., Haller-Kikkatalo K., Oras A., Roos K., Kirss A., Uibo R. (2022). Plasma cytokines during pregnancy provide insight into the risk of diabetes in the gestational diabetes risk group. *Journal of Diabetes Investigation*.

[31] Yousif A. F., Al-Salih R. M., Al-Naser A. H. (2021). Association between gestational diabetes and proinflammatory cytokine (IL-1*β*). *Indian Journal of Forensic Medicine & Toxicology*.

[32] Hou M., Li F. (2020). Changes of intestinal flora, cellular immune function and inflammatory factors in patients with gestational diabetes mellitus. *Acta Medica Mediterranea*.

[33] Friebe-Hoffmann U., Antony L., Kruessel J. S., Pawlowski B., Hoffmann T. K. (2017). Peripheral immunological cells in pregnant women and their change during diabetes. *Experimental and Clinical Endocrinology and Diabetes*.

[34] Yang Y., Liu, Liu B. (2018). Functional defects of regulatory T cell through interleukin 10 mediated mechanism in the induction of gestational diabetes mellitus. *DNA and Cell Biology*.

[35] Pendeloski K. P. T., Mattar R., Torloni M. R., Gomes C. P., Alexandre S. M., Daher S. (2015). Immunoregulatory molecules in patients with gestational diabetes mellitus. *Endocrine*.

[36] Moreli J. B., Corrêa-Silva S., Damasceno D. C. (2015). Changes in the TNF-alpha/IL-10 ratio in hyperglycemia-associated pregnancies. *Diabetes Research and Clinical Practice*.

[37] Zhuang Y., Zhang J., Li Y. (2019). B lymphocytes are predictors of insulin resistance in women with gestational diabetes mellitus. *Endocrine, Metabolic and Immune Disorders-Drug Targets*.

[38] Noureldeen A. F. H., Qusti S. Y., Al-Seeni M. N., Bagais M. H. (2014). Maternal leptin, adiponectin, resistin, visfatin and tumor necrosis factor-alpha in normal and gestational diabetes. *Indian Journal of Clinical Biochemistry*.

[39] Schober L., Radnai D., Spratte J. (2014). The role of regulatory T cell (Treg) subsets in gestational diabetes mellitus. *Clinical and Experimental Immunology*.

[40] Kuźmicki M., Telejko B., Lipińska D. (2014). Stężenie interleukiny-6, receptora dla interleukiny-6 i glikoproteiny 130 oraz cytokin zależnych od limfocytów Th17 u pacjentek z cukrzycą ciążową. *Endokrynologia Polska*.

[41] Gueuvoghlanian-Silva B. Y., Torloni M. R., Mattar R. (2012). Profile of inflammatory mediators in gestational diabetes mellitus: phenotype and genotype. *American Journal of Reproductive Immunology*.

[42] Abdel Gader A. G. M., Khashoggi T. Y., Habib F., Awadallah S. B. A. (2011). Haemostatic and cytokine changes in gestational diabetes mellitus. *Gynecological Endocrinology*.

[43] Wang Q., Pan M., Zhang T. (2022). Fear stress during pregnancy affects placental m6A-modifying enzyme expression and epigenetic modification levels. *Frontiers in Genetics*.

[44] Siddiqui S., Waghdhare S., Jhaa S., Dubey S. (2019). Role of immunological markers in gestational diabetes mellitus-a brief review. *Diabetes & Metabolic Syndrome*.

[45] Aune D., Sen A., Henriksen T., Saugstad O. D., Tonstad S. (2016). Physical activity and the risk of gestational diabetes mellitus: a systematic review and dose-response meta-analysis of epidemiological studies. *European Journal of Epidemiology*.

[46] West N. A., Crume T. L., Maligie M. A., Dabelea D. (2011). Cardiovascular risk factors in children exposed to maternal diabetes in utero. *Diabetologia*.

[47] Tam W. H., Ma R. C. W., Yang X. (2008). Glucose intolerance and cardiometabolic risk in children exposed to maternal gestational diabetes mellitus in utero. *Pediatrics*.

[48] Harlev A., Wiznitzer A. (2010). New insights on glucose pathophysiology in gestational diabetes and insulin resistance. *Current Diabetes Reports*.

[49] Chiefari E., Arcidiacono B., Foti D., Brunetti A. (2017). Gestational diabetes mellitus: an updated overview. *Journal of Endocrinological Investigation*.

[50] Olefsky J. M., Glass C. K. (2010). Macrophages, inflammation, and insulin resistance. *Annual Review of Physiology*.

[51] Odegaard J. I., Chawla A. (2013). Pleiotropic actions of insulin resistance and inflammation in metabolic homeostasis. *Science*.

[52] Lobo T., Borges C., Mattar R. (2018). Impaired Treg and NK cells profile in overweight women with gestational diabetes mellitus. *American Journal of Reproductive Immunology*.

[53] Gomes C., Torloni M., Gueuvoghlanian-Silva B., Alexandre S., Mattar R., Daher S. (2013). Cytokine levels in gestational diabetes mellitus: a systematic review of the literature. *American Journal of Reproductive Immunology*.

[54] Basu J., Datta C., Chowdhury S., Mandal D., Mondal N., Ghosh A. (2020). Gestational diabetes mellitus in a tertiary care hospital of Kolkata, India: prevalence, pathogenesis and potential disease biomarkers. *Experimental and Clinical Endocrinology & Diabetes*.

[55] Pantham P., Aye I., Powell T. (2015). Inflammation in maternal obesity and gestational diabetes mellitus. *Placenta*.

[56] Syngelaki A., Visser G., Krithinakis K., Wright A., Nicolaides K. (2016). First trimester screening for gestational diabetes mellitus by maternal factors and markers of inflammation. *Metabolism*.

[57] Zhang J., Chi H., Xiao H. (2017). Interleukin 6 (IL-6) and tumor necrosis factor *α* (TNF-*α*) single nucleotide polymorphisms (SNPs), inflammation and metabolism in gestational diabetes mellitus in inner Mongolia. *Medical Science Monitor*.

[58] Amirian A., Mahani M., Abdi F. (2020). Role of interleukin-6 (IL-6) in predicting gestational diabetes mellitus. *Obstetrics & Gynecology Science*.

[59] Mirabelli M., Tocci V., Donnici A. (2023). Maternal preconception body mass index overtakes age as a risk factor for gestational diabetes mellitus. *Clinical Medicine*.

[60] Abell S., De Courten B., Boyle J., Teede H. (2015). Inflammatory and other biomarkers: role in pathophysiology and prediction of gestational diabetes mellitus. *International Journal of Molecular Sciences*.

[61] Parrettini S., Caroli A., Torlone E. (2020). Nutrition and metabolic adaptations in physiological and complicated pregnancy: focus on obesity and gestational diabetes. *Frontiers in Endocrinology*.

[62] Sharma S., Banerjee S., Krueger P. M., Blois S. M. (2022). Immunobiology of gestational diabetes mellitus in post-medawar era. *Frontiers in Immunology*.

